# An Embedded Electromyogram Signal Acquisition Device

**DOI:** 10.3390/s24134106

**Published:** 2024-06-24

**Authors:** Changjia Lu, Xin Xu, Yingjie Liu, Dan Li, Yue Wang, Wenhao Xian, Changbing Chen, Baichun Wei, Jin Tian

**Affiliations:** 1 China Coal Research Institute, Beijing 100013, China; 2 Emergency Science Research Academy, China Coal Research Institute, Beijing 100013, China; 3 School of Medicine and Health, Harbin Institute of Technology, Harbin 150001, China; 4 School of Mechatronics Engineering, Harbin Institute of Technology, Harbin 150001, China

**Keywords:** exoskeleton, surface electromyography, embedded device, signal preprocessing, intention recognition

## Abstract

In this study, we design an embedded surface EMG acquisition device to conveniently collect human surface EMG signals, pursue more intelligent human–computer interactions in exoskeleton robots, and enable exoskeleton robots to synchronize with or even respond to user actions in advance. The device has the characteristics of low cost, miniaturization, and strong compatibility, and it can acquire eight-channel surface EMG signals in real time while retaining the possibility of expanding the channel. This paper introduces the design and function of the embedded EMG acquisition device in detail, which includes the use of wired transmission to adapt to complex electromagnetic environments, light signals to indicate signal strength, and an embedded processing chip to reduce signal noise and perform filtering. The test results show that the device can effectively collect the original EMG signal, which provides a scheme for improving the level of human–computer interactions and enhancing the robustness and intelligence of exoskeleton equipment. The development of this device provides a new possibility for the intellectualization of exoskeleton systems and reductions in their cost.

## 1. Introduction

As an important tool to assist human movement, exoskeleton equipment brings new hope and possibilities for the enhancement of individual body functions. With the gradual maturation of exoskeleton technology, its application has been extended from the military field to industrial and medical fields. However, the intelligence of current exoskeleton devices is often limited by their insufficient recognition of the user’s motion intention, resulting in a delay between the motion of the device and the user’s motion, and it is difficult to achieve real-time and efficient assistance.

In recent years, with the rapid development of EMG research [1], researchers have found new directions in recognizing human motion intent [2,3]. By collecting and utilizing human EMG signals, users’ motion intentions can be more accurately captured, thus achieving the optimization and real-time control of exoskeletal devices [4,5,6]. Some research has already used EMG for exoskeleton control and improved the human–computer interaction ability of exoskeletons, but most studies still fail to use EMG in exoskeleton robots. Although EMG technology is relatively mature and efficient, its application in exoskeletons is limited due to the large size and high cost of acquisition devices. However, the conditions for the acquisition of electromyographic signals are slightly harsh, the accurate acquisition of electromyographic signals takes a long time, and ordinary electrode patches are difficult to bear. In recent years, flexible electrode patches have become a solution to this problem. In this study, an embedded EMG acquisition device is designed to collect and utilize EMG completely, reduce development costs and equipment volume, and thus improve the integration degree of exoskeletons [7,8]. This device can improve the level of human–computer interactions and the intelligence of the exoskeleton at the same time, reduce the development costs of exoskeleton equipment, promote the entry of exoskeleton equipment into the affordable market, and provide help to more users.

**Figure 1:**
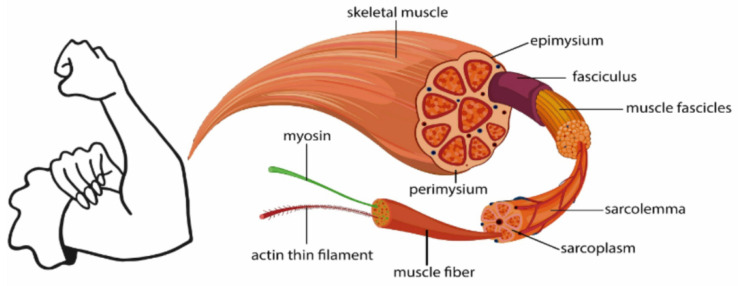
Schematic diagram of surface electromyography signals.

Human action potentials are formed mainly by sodium, calcium, and potassium ions passing through cell membranes. Each muscle contraction and diastole produces a potential difference, and the superposition of active electrode units is the bioelectrical signal, while the myoelectric signal is an easy-to-collect bioelectrical signal [9]. This is shown in Figure 1.

The acquisition of electromyography signals can be divided into surface electromyography signal and intramuscular electromyography signal acquisition. Considering that wearing exoskeleton devices requires frequent activities and long-term wear and that intramuscular EMG signals may cause damage to users, this study chooses to collect surface EMG signals [10]. Surface EMG signals contain rich information about the human body and they can be generated before movement of the human body. When the human body moves, surface signals are generated 30–150 ms before the limb moves. Therefore, if surface EMG signals can be accurately collected and used in an exoskeleton device, synchronous movement of the exoskeleton and the human body can be realized, and the fluency and intelligence of the device can be enhanced.

Surface EMG signals [11,12] usually have three characteristics: The first characteristic is that they have a low frequency; the main energy is concentrated at 20–150 Hz, and the overall range is 0–500 Hz. The second characteristic is their weakness; the amplitude is generally between 0 and 10 mV. The third characteristic is high impedance; the surface resistance of the human body is not constant, and the different humidities and temperatures of the skin lead to changes in resistance of up to 450 kΩ. Because of these characteristics, it is difficult to simply and directly collect EMG signals. At the same time, cross-talk interference between muscles should be paid attention when collecting surface EMG signals through multiple channels [13,14]. Therefore, circuit design and device selection should be particularly studied in embedded design.

In summary, this paper is devoted to the study of acquiring surface EMG signals in lower-extremity exoskeleton equipment at low cost and in a small volume, so as to realize more intelligent and accurate motion intention recognition. The equipment design has the following characteristics:

(1) The device has a low cost, small size, and strong compatibility: a total of eight channels of surface EMG signals can be collected. The data output can be transmitted to the host computer in real time, the waveform display can be adjusted by the tester, and data analysis and calibration can be compiled online. The reserved interface can be designed to control the external skeleton.
(2) Wired transmission mode: Exoskeletons have a variety of application scenarios, especially in medical, industrial, and military fields, but the magnetic field environment of some scenes is complex, or there is a need for shielded signals. In view of the packaging requirements of exoskeleton devices, for the upload method of the embedded surface EMG, wireless methods such as Bluetooth and WiFi modules are disregarded, and USB synchronous wired transmission is chosen to ensure the application of the device in various scenarios.
(3) Integrated signal processing circuit: The embedded module is designed to adjust the amplification and sampling frequency of the surface EMG signal. Through the integrated design of the circuit and the program compilation of each module, the sampling data are pre-processed using a noise reduction filter and envelope, and the signal-to-noise ratio is improved. An ADS1298 chip (Texas Instruments production, purchased in Beijing, China) can adjust the magnification and sampling frequency to realize the acquisition of surface EMG signals on demand.

The device solves the problems of high cost and large volume of surface EMG acquisition at present. In the embedded surface electromyographic signal acquisition device designed in this paper, part of the data analysis function of the precise electromyographic signal sensor is disregarded, aiming to replace the role of the large-volume electromyographic sensor in the acquisition of electromyographic signals on the exoskeleton device; reduce the development cost of electromyographic signals in the exoskeleton device; and promote the development of exoskeleton technology in the direction of intelligence, digitalization, and sustainability.

## 2. Low-Cost Surface EMG Signal Acquisition Module Design

**Figure 2:**
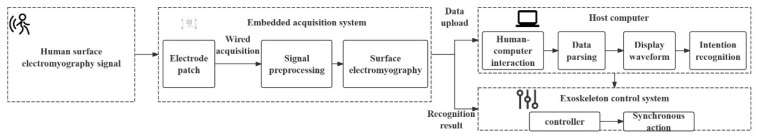
Overall design framework of embedded surface EMG acquisition device.

The whole frame of the device is divided into three parts: a surface EMG acquisition device, an upper computer, and an exoskeleton control system. A medical silver fiber electrode patch is used for signal acquisition, and eight-channel signal acquisition is designed with two electrode patches per channel. When bipolar electrodes are applied to small muscles, the electrode spacing should not exceed one-quarter of the muscle fiber length to avoid unstable recording caused by tendon and motion end-plate effects; thus, the electrode spacing is set to 20 mm. The device adjusts the signal through the hardware, including the signal magnification and sampling frequency [15]. Through USB synchronous wired transmission, data are stored and analyzed in the upper computer, the waveform is displayed, and other operations are carried out through an algorithm to achieve human movement identification so as to control the exoskeleton device and achieve synchronous action. The specific framework is shown in Figure 2.

### 2.1. Hardware Layer Design Framework

**Figure 3:**
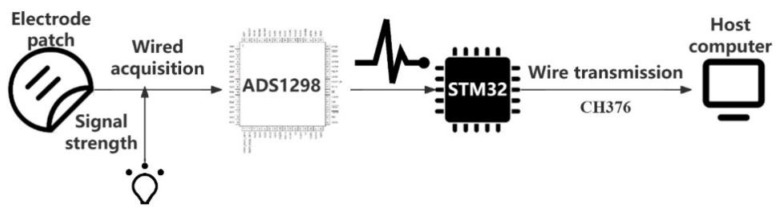
Hardware design framework of embedded surface EMG acquisition device.

The design circuit of the device includes an amplifier circuit, a protection circuit, a filter circuit, a power supply circuit, and other basic circuits. The device increases the original signal output circuit, and this can be divided into two circuits by adjusting the amplification of the device: one carries out adjustable amplification processing, and the other adjusts the magnification to 1, which is used as the next circuit to collect the original signal. Data are then transmitted to the exoskeleton. The surface EMG signal of the selected muscle group is collected by the silver fiber electrode patch, and the original signal is collected using the wired collection method. The specific design of the hardware layer is shown in Figure 3.

### 2.2. Hardware Level Design

As the main way to collect physiological signals, an electrode patch is widely used in the medical field, and it is suitable for the accurate collection of physiological signals such as EEG, myoelectric, and ECG signals. Its design is small, convenient, low cost, and researcher friendly. The device uses a silver fiber electrode patch to collect human surface electromyography, which is in line with medical standards. Silver material has a certain insulation effect that reduces environmental interference. Before collecting signals, the skin needs to be treated, such as rubbing with alcohol and removing hair.

**Figure 4:**
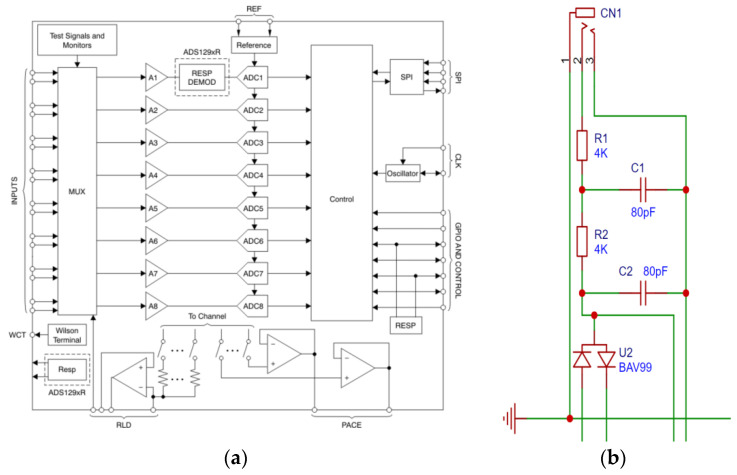
Screenshot of several circuits. (**a**) Schematic diagram of ADS1298 circuit structure. (**b**) Circuit design of second-order passive filter.

The system uses an ADS1298 high-precision ADC for digital conversion. The ADS1298 chip is an analog-to-digital converter (ADC) that integrates eight low/zero-noise programmable gain amplifiers and eight high-resolution analog-to-digital converters. The chip is designed to detect EEG signals with low power consumption and has a highly programmable multiplexer that can use any channel as a reference channel to set multiple gain values. The chip sampling rate can range from 250 sps (samples per second) to 32 Ksps. The chip has an integrated test circuit, a gain amplification circuit, full differential input, a right-leg drive circuit, a controller and SPI communication interface, and other circuits. The selection of ADS1298 [16] can solve the problems of simultaneous acquisition of multiple signals [17], low frequency, weakness, and high impedance in surface EMG; meet the requirements of a compact and convenient exoskeleton; reduce the size of the circuit board; and reduce the total power consumption and development cost of the system. Its internal gain amplifier PGA can be set to magnifications of 1, 2, 3, 4, 6, 8, and 12. A diagram of the internal basic structure of the chip is shown in Figure 4a.

A second-order passive low-pass filter is used in the acquisition front end to remove some high-frequency noise. The design is shown in Figure 4b. The cut-off frequency is set to 500 Hz. Then, the selection of resistance and capacitance is determined according to transfer functions (1) and (2), where R_1 = R_2 = R, C_1 = C_2 = C. After calculation, it is determined that the design R = 4 KΩ and C = 80 Pf can meet the requirements of 500 Hz. At the same time, a voltage regulator diode circuit is added to protect the circuit board when the voltage is too high. The main function of the right-leg drive circuit in the chip is to feed back the common-mode signal in the measurement system to the right leg of the measured person so as to reduce the potential difference between the measurement circuit and the reference potential, as well as improve the common-mode rejection ratio. To reduce interference from the operating frequency, especially 50/60 Hz noise from the power cord, it is set as a reference circuit when collecting the EMG signal, especially when collecting the lower-extremity surface EMG signal, which maximizes its use. A second-order passive low-pass filter is also included in the design. CLKSEL uses an internal 2 M clock and only needs to be connected to a power source. The power driver module adopts the 5.0 V and 3.3 V voltage supply modes, a depressed-voltage regulator link, and ordinary LDO components to obtain a voltage regulator power supply of 5.0 V. The rest of the circuit is built according to the classic circuit in the user manual, ensuring that the common-mode rejection ratio is not less than 80 dB, the amplifier magnification is 6, the internal noise is not greater than 50 μVpp, and the input impedance is above 15 MΩ to obtain a clear signal.

(1) $$H\left(j\omega \right)=\frac{1}{\left(1-\omega^{2}R^{2}C^{2}\right)+3j\omega RC}=\left|H\left(j\omega \right)\right|∠\theta (w)$$

(2) $$\left|H\left(j\omega \right)\right|=\frac{1}{\sqrt{{\left(1-\omega^{2}R^{2}C^{2}\right)}^{2}+9\omega^{2}R^{2}C^{2}}}$$

**Figure 5:**
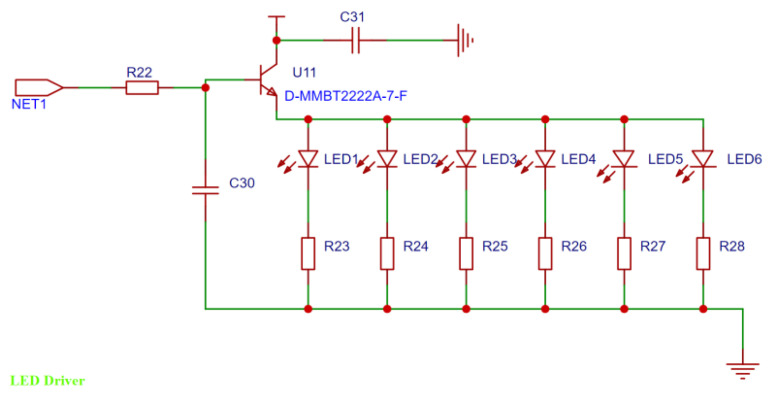
LED driver circuit.

The master selected STM32F405RGT6 (ST (STMicroelectronics) output, purchased in Shanghai, China) is a high-performance ARM Cortex-M4 MCU, with resources such as a 1 MB Flash, 168 MHz CPU, ART accelerator, and watchdog. It is compact and flexible, has a fast computing speed, supports a variety of peripheral combinations, and is suitable for exoskeleton design needs. The main control chip communicates with ADS1298 through the SPI interface to collect surface EMG signals. An external crystal oscillator is used as the clock source to ensure the stable performance of the device. The LED module is designed with eight channels. PB8 to PB13 of STM32F405RGT6 control channels 1 to 6, and PB0 and PB1 are reserved to control channels 7 and 8, respectively. Six LEDs are lit in turn according to the signal strength, thus indicating signal strength detection. The design is shown in Figure 5, and it is used for intuitive signal acquisition and display during the test stage and subsequent use.

Finally, a CH376 chip (made in WCH (Nanjing Qinheng Microelectronics Co., Ltd., purchased in Nanjing, China) is used to realize the data transmission function between the lower computer and the upper computer. The CH376 chip has a variety of data communication interfaces, internal integrated communication protocol firmware, and a self-check function to achieve circuit protection. In exoskeleton applications, the CH376 chip not only has a low-power operating mode but can also maximize space utilization and improve utilization efficiency. The device uses the classic 8-bit parallel port mode to communicate with STM32, and the maximum communication rate can reach 2 MB/s.

### 2.3. Software Layer Design Framework

**Figure 6:**
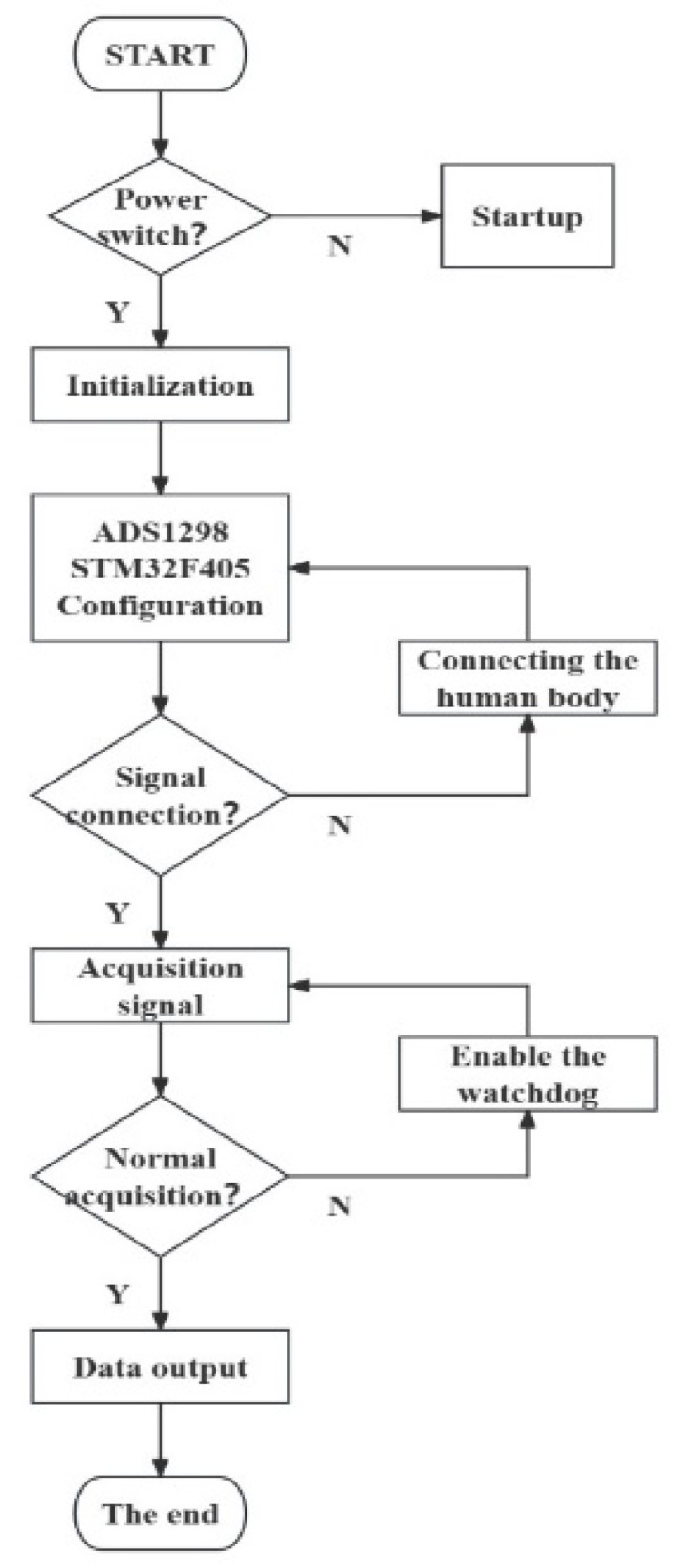
Software design process framework.

The device is designed with full consideration of portability, and it is easy to operate and use in various exoskeleton devices. The code is written in C++ language (version C++20), while the upper computer software is developed in Python language (version 3.11.2). Software design is mainly divided into two parts: lower computer operation control and upper computer signal display. The operation control of the lower machine includes ADS1298, the STM32 module, the LED driver module, the power module, the data transmission module, watchdog, and other parts of code writing and burning that are employed to control each module. An overall flow diagram is shown in Figure 6. A human–machine interface is designed for the upper computer signal display, which is used to complete the filtering processing of the surface EMG signal, display the signal of each channel, and perform simple data analysis. In practical applications, the main function of the UI is to assist personnel in debugging operating equipment, so the design emphasizes simplicity, clarity, and high operability and minimizes focus on beautifying the UI.

### 2.4. Software Layer Design

First, the lower machine is initialized, and it is configured by modifying the internal registers of ADS1298. After successful initialization, the main program detects the lithium battery level, and, when the battery is low, it will issue an alarm through a preset red LED light. The main program then enters the main loop and records the surface EMG signal collected by ADS1298. STM32 receives data through the SPI mode and writes to the data buffer. During the test phase, the data are uploaded to the software of the upper computer through the USB interface for an analysis. STM32 receives not only the eight-channel ADS1298 EMG data but also the 3-byte register status control word containing the electrode connection information. By judging the status control word, the single-chip computer can detect the situation of electrode shedding, and, once electrode shedding is detected, it will send an alarm signal. When storing data on the upper computer, the eight channels of data are separated with commas to facilitate direct data processing. External input instructions are used to test whether the chip can burn the code normally and ensure the operation rate of the code to be burned.

After initializing ADS1298, the internal crystal excitation is selected as the working main frequency, CLKSEL and RESET are set to 1, and crystal vibration is awaited. Then, the pin START is configured to 1 to activate the device, and it is put into read/write mode. The other adjustable parameters of ADS1298 are set as follows: the magnification is 6, and the acquisition frequency is 1 KHz. ADS1298 communicates with STM32 through SPI and follows protocol standards for read and write operations. The SPI interface contains four signals: CS, SCLK, DIN, and DOUT. The interface reads and writes registers to control the operation of ADS1298. The DRDY output is used as a status signal indicating when the data are ready.

STM32 needs to receive and store signals, preprocess them [18], and upload data. The main tasks are to filter and envelope the collected EMG signals. In this study, a third-order Butterworth bandpass filter is selected, and the passband cutoff frequency is 25 Hz to 400 Hz. At the same time, LED lights are controlled to indicate the strength of the signal. As the signal gradually increases, six LED lights are lit in turn to indicate the signal strength and continue to output the processed signal. In addition, STM32 controls the operation of the other devices.

The key to using the CH376 chip to realize data transmission is to use its built-in communication protocol firmware and a variety of data communication interfaces. The device uses the classic 8-bit parallel port mode to communicate with STM32, and the maximum communication rate can reach 2 MB/s. During the initialization process, the register of the CH376 chip is configured to set the communication parameters. The main operations include the following: the reset pin is set to 1 to ensure that it enters the initial state, then the communication mode is set, CH376 is set to 8-bit parallel port mode, and the relevant communication parameters are configured. The baud rate is set to 9600 in this experiment. The communication mode is initiated by the control signal so that it is ready to receive and send data. STM32 transmits data with CH376 through the SPI interface, and it sends data to the host computer through CH376. In addition to normal transmission, the CH376 self-check function is used to detect possible errors during transmission and perform data retransmission.

In addition, the device enables the built-in watchdog function of STM32F405RGT6 for the security monitoring of the system to prevent the system from entering a dead loop or unrecoverable errors. The clock is connected to the external crystal oscillator, the watchdog timeout time is set to 2 min, and the watchdog is started to monitor the running state of the system. In the main program, the watchdog should be fed regularly to avoid the timeout reset of the watchdog, and the system status should be periodically detected to determine whether the reset operation is necessary.

### 2.5. Test and Inspection

The device was used to collect human lower-limb signals [19] and test the collection results. When the number of muscles selected reaches five, skeletal muscle can be used for feature classification to reach a stable value; when the number reaches nine, the accuracy of feature classification begins to decline [20,21]. In order to reduce the error caused by muscle duplication, after studying the muscle activities related to the selected movements, the device was used to collect data on six selected muscles, namely, the biceps femoris, lateral femoris, medial femoris, tibialis anterior, gastrocnemius, and semitendinosus muscles of the human lower limbs, as the source of surface EMG [22,23]. These muscles are used to fix and stretch the bones in order to realize the action of the lower limbs, mainly involving the ankle, knee, and hip joints. Considering that surface EMG signals are characterized by weakness and a low main frequency, in order to reduce the influence of interfering factors, the surface of the selected muscle groups was cleaned, and 75% alcohol wipes were used to wipe the surface before the electrode patch was applied to the subject [24].

Aging leads to the loss of muscle density and natural growth of the human body, with peak muscle mass reached at about 25 years of age. In this experiment, a 25-year-old male subject with a height of 174 cm and weight of 72 kg, at an ambient temperature of 26 °C, was selected for signal collection and verification. A silver fiber electrode patch was used for testing to prevent interference from external environmental magnetic field factors. The selected materials all met the medical standards for devices in the medical field, which is convenient for the subsequent design of reusable electrode patches. Before the experiment, the surface of the legs of the subject was cleaned with alcohol. Because the subject did not have much hair, hair removal was not carried out. A six-channel EMG acquisition and development board was used to collect EMG signals, including the simple actions of straight-line walking, turning walking, sitting up, and squatting, forming one group of data. A total of five groups of data were collected, with each being collected successively. To reduce the effects of muscle fatigue, a 3–5 min rest was taken between each movement, with a 10 min interval between each set of movements. The main reasons for selecting the actions [25,26] were as follows:

(1) Normal standing: For this movement, the surface EMG signal was collected under normal circumstances, recorded as a static signal, and compared with the data collected from other actions.
(2) Linear walking [27]: For this movement, the average walking speed of adults of 1.2 m/s was taken as the standard, and the signal collection time was about 5 s. Considering that normal walking involves the most movement of the legs, this process mainly causes regular changes in the hip, knee, and ankle joints.
(3) Sitting up: In this movement, the lateral femoris muscle, biceps femoris muscle, semitendinosus muscle, tibialis anterior muscle, and other muscles are mainly used to drive the action produced by the joint; there is no gait cycle; and it involves joint movement that is not involved in turning walking, mainly the bending/extension of the hip and knee joint.
(4) Squat: This movement is the next step of sitting up, causing knee flexion and ankle dorsiflexion.

According to the selection of the hardware part of the design device, EMG signal collection needed to be carried out under the following conditions:

(1) Ambient temperature of 10~70 °C; (2) input signal common-mode rejection ratio ≥ 80 dB; (3) power supply voltage of 5 V; (4) input impedance ≥ 15 MΩ; (5) electrode spacing not exceeding one-quarter of the muscle fiber length to avoid unstable recording caused by tendon and motor end-plate effects; and (6) skin kept as dry as possible.

**Figure 7:**
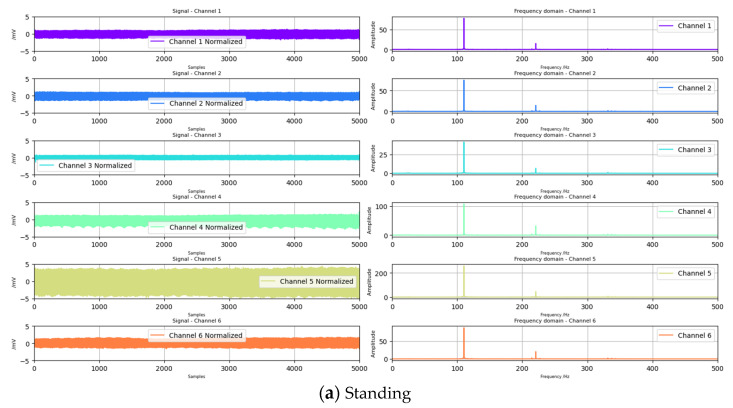

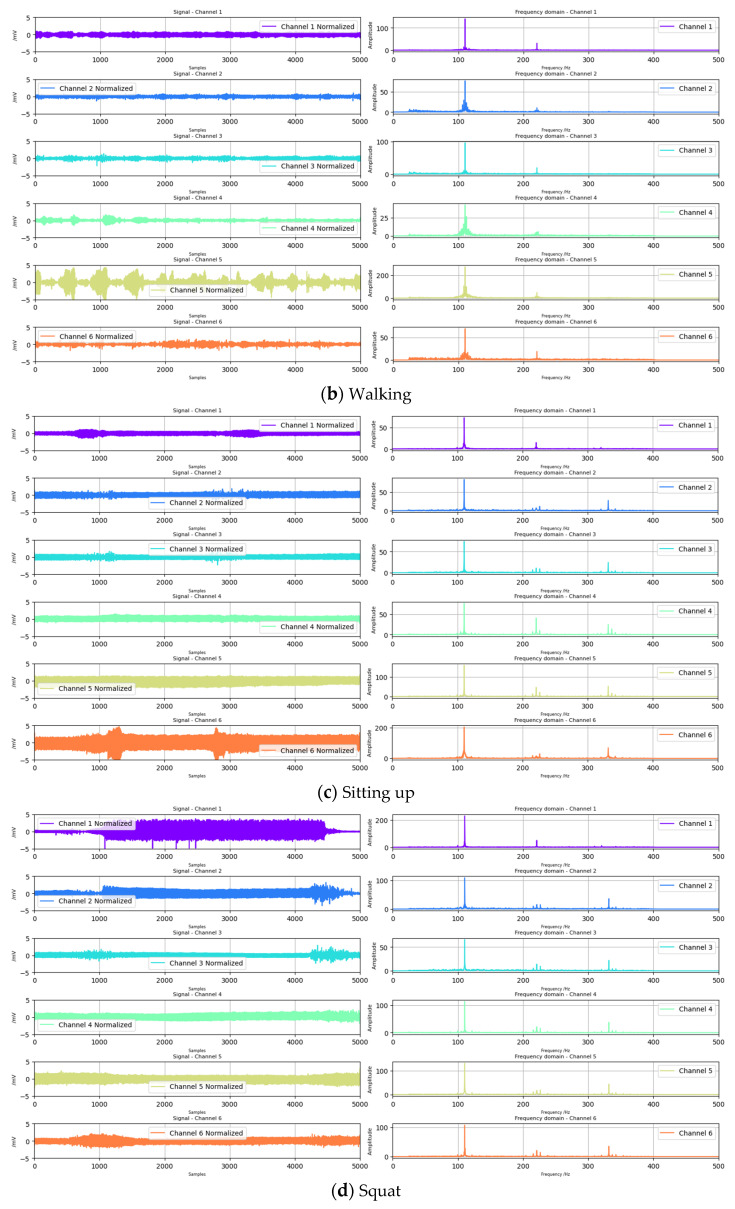
Partial data display after filtering.

The collected raw data were enveloped in the upper computer and processed by a third-order Butterworth bandpass filter with a frequency range of 25 Hz to 400 Hz. The collected signal was within 0~500 Hz, which is in accordance with the characteristics of EMG. After filtering, the experimental data were visualized, as shown in the following figure (Figure 7), where the left side shows a signal plot, and the right side shows a spectrum diagram.

The experimental data of the biceps femoris are taken as an example and compared with JJF 1896–2021 issued by the National Metrology Technical Specification of the People’s Republic of China, Calibration Specification for Electromyographs and Evoked Response Equipments [28]. The data results are shown in Table 1, wherein the calculation formulas of the parameters are as follows:

(3) $$\delta_{H}=\frac{H_{x}-H_{0}}{H_{0}}\times 100\%$$

Formula:

$\delta_{H}$—Waveform amplitude indication error;
$H_{x}$—Waveform amplitude measurement, mV or μV;
$H_{0}$—Standard value of waveform amplitude, $H_{0}\;$=$\;V_{0}$/K (where $V_{0}$ is the amplitude of the signal generator output waveform, and K is the attenuation ratio of the balance attenuator, with a theoretical value of 1000), mV or Μv.

(4) $$\delta_{f}=\frac{f_{x}-f_{0}}{f_{0}}\times 100\%$$

Formula:

$\delta_{f}$—Waveform frequency indication error;
$f_{x}$—Waveform amplitude measurement, Hz;
$f_{0}$—Standard value of waveform amplitude, Hz.

(5) $$CMRR=20lg\frac{U_{i}}{U_{o}}$$

Formula:

CMRR—Common-mode rejection ratio, dB;
$U_{i}$—Input signal peak-to-peak value, V;
$U_{o}$—Voltage amplitude measurement, V.

As shown in the table above, the waveform amplitude, waveform frequency, and amplitude–frequency characteristics are all in line with the standard, but the accuracy still needs to be improved. The remaining five channels, namely, the lateral femoris, medial femoris, tibialis anterior, gastrocnemius, and semitendinosus muscles, are consistent, and further standardized signal processing is required [29,30]. However, the overall CMRR of the device is only greater than 80 dB, so it is necessary to further improve the CMRR. The filtered signal has a high signal-to-noise ratio and can accurately reflect the changes in the EMG signal. At the same time, the system can collect and process six-channel EMG signals in real time to meet the needs of real-time control for exoskeleton equipment. Future research can further optimize the signal processing algorithm, improve the intelligence level of the system, and verify it in actual exoskeleton devices.

## 3. Results

The device was designed with the aims of reducing the cost of EMG acquisition, reducing the volume of the device, and realizing partial analyses of EMG so as to be applied in exoskeleton equipment to assist in identifying the intention of human movement and enhance the degree of human–computer interactions. By explaining the hardware and software of the equipment, this paper describes the designed electromyographic signal acquisition device, and, after testing, it is found that a complete and clear surface electromyographic signal is successfully displayed in the upper computer, which meets the use requirements and basically meets the national standard of electromyographic signal acquisition equipment.

Compared with existing EMG sensors on the market, as shown in Table 2, after reducing some performance parameters, the demand for low cost and low volume is met.

However, the device still has the possibility of further development:

(1) The ADS1298 chip supports cascading or daisy-chain connection, which can meet the need of more channels in surface EMG acquisition. Depending on the muscle selected, by increasing the number of channels, the accuracy of signal acquisition can be further improved; thus, the intention of human movement can be more accurately captured and analyzed, and the performance of the exoskeleton device can be improved.
(2) If wired transmission is not suitable, the CH376 chip can be replaced with Bluetooth or wireless WiFi and other modules. The wired mode selected in this study is mainly used in special environments, such as coal mines, confidential places that cannot use WiFi, and computer rooms.
(3) The method used in this paper to deal with EMG only uses the simplest processing flow. If more accurate processing is needed, it can be designed according to requirements and integrated into the single-chip microcomputer to achieve more convenient EMG processing.

In general, the embedded EMG acquisition device designed in this paper has the possibility of mass production, but the device is in the early stage of development, with the aims currently being to achieve the overall requirements, reduce the cost and volume, and achieve greater compatibility. However, there are still many areas that can be improved. Below, this study makes suggestions on how to improve the available lines of the equipment.

## 4. Future Outlook

In recent years, the research and development of exoskeleton machines has become increasingly diversified, but the two main aspects of the overall development trends are flexible wearable materials and human–computer interaction intelligence. The use of surface EMG makes the two related, and the successful development of this device is expected to further promote the development of exoskeleton devices. However, there are still some shortcomings in the acquisition equipment, so this paper makes the following suggestions for the future use of the equipment:

1. Flexible wearable electrode: The embedded device uses an electrode patch for signal acquisition. Although the electrode patch is cheap, it is a disposable commodity and cannot be reused, making it inconvenient. With the development of flexible wearable devices, researchers can design a flexible wearable surface EMG acquisition device or use a flexible circuit board for signal acquisition design to improve the convenience and user experience of the device.
2. Integrated touch screen: Since this device is designed for exoskeleton equipment, if the funds are sufficient, the PC can be skipped, and a touch screen design can be added to the PCB circuit board, or the display screen can be connected to the exoskeleton device packaging panel so as to realize equipment inspection without disassembly and improve the convenience and real-time operation.
3. Power life management: The exoskeleton itself consumes a lot of power, and the complex algorithms used to fully recognize the human EMG also increase battery loss. When the same action is performed for a long time without any change, it is hoped that the acquisition device can enter the sleep state to reduce battery loss. The use time of the device should be increased so as to fit the daily needs of the human body.

Through the above improvements, the device is expected to play a greater role in the application of exoskeleton equipment, promote the development of exoskeleton technology, and enhance the intelligence and practicality of human–computer interactions. In the future, with the continuous progress of technology and the increase in application demand, the device will be continuously optimized and upgraded to provide strong support for the realization of more intelligent and efficient exoskeleton equipment.

## Figures and Tables

**Table 1:** Comparison between collected data and national standards.

	JJF 1896–2021	Standing	Walking	Sitting Up	Squat
Waveform amplitude	100 μV~5 mV, ±10%	3.138 mV	2.110 mV	2.697 mV	8.750 mV
Waveform frequency	1 Hz~3 kHz, ±5%	Conforms to	Conforms to	Conforms to	Conforms to
Common-mode rejection ratio	≥110 dB	85.3 dB	86.1 dB	84.4 dB	94.5 dB
Amplitude–frequency characteristic	With 10 Hz sine wave as the reference value, it varies with frequency in the range of 1~3 kHz, and the amplitude deviation is +5~−30%	Conforms to	Conforms to	Conforms to	Conforms to

**Table 2:** Partial product comparison.

Company Product	Overall Dimensions	Price	Performance Parameters
Device designed in this paper	About 15 × 15 × 5 cm	USD 275.93	8 channels Sample rate: 16 Ksps (MAX) Connection mode: USB synchronous cable transmission Common-mode rejection ratio: >80 dB
Menovomed	About 200 × 150 × 100 mm	USD 24,833.7	8 channels Sampling rate: 4000 sps (MAX) Connection mode: WI-FI 5G SNR: >95 dB Input impedance: >10 MΩ
Delsys	About 210 × 120 × 150 mm	USD 27,592.6	16 channels Sample rate: 4370 sa/s Connection mode: proprietary RF protocol
Noraxon	About 261 × 36 × 29 mm	USD 35,868.5	16 channels Sampling rate: 4000 sps (MAX) Connection mode: proprietary RF protocol Common-mode rejection ratio < −100 dB Best signal-to-noise ratio
PPB-Ergo	About 85 × 54 × 10 mm	USD 27,591.15	4 channels Sampling rate: 4000 sps (MAX) Connection mode: Bluetooth Class II Common-mode rejection ratio: 100 dB

## Data Availability

The data presented in this study are available on request from the corresponding author due to the data in this paper were only used by the authors’ validation equipment.
